# Evidence of abnormal scalar timing property in alexithymia

**DOI:** 10.1371/journal.pone.0278881

**Published:** 2023-01-23

**Authors:** Carmelo Mario Vicario, Vito Scavone, Chiara Lucifora, Alessandra Falzone, Giovanni Pioggia, Sebastiano Gangemi, Giuseppe Craparo, Gabriella Martino

**Affiliations:** 1 Dipartimento di Scienze Cognitive, Psicologiche, Pedagogiche e degli studi culturali, Università di Messina, Messina, Italy; 2 ISTC-CNR, Rome, Italy; 3 Institute for Biomedical Research and Innovation (IRIB), National Research Council of Italy (CNR), Messina, Italy; 4 Dipartimento di Medicina Clinica e Sperimentale, Università di Messina, Messina, Italy; 5 Faculty of Human and Social Sciences, Kore University of Enna, Cittadella Universitaria, Enna, Italy; Liverpool John Moores University, UNITED KINGDOM

## Abstract

Evidence suggests that incidental modulation of affective states affects the ability to keep track of time. Alexithymia represents an ideal condition to further address the emotion-time processing link, as it refers to a trait characterized by a deficit of affective processing. 31 healthy participants completed an online version of the TAS-20 scale, which measures alexithymia, and a time reproduction task of visual stimuli related to positive (i.e., happiness) and negative (i.e., anger) facial expressions. Results documented a positive correlation between TAS-20 score and the variability in reproducing sub-second durations of the anger expression stimuli We also found an overestimation of sub-second durations of non-affective expressions in borderline/alexithymic participants. Finally, in line with the literature, we confirmed the overall tendency to overestimate the duration of anger expression stimuli. These findings, which can be interpreted in terms of abnormal scalar timing property in alexithymia, expand previous investigations linking this personality trait with abnormal processing of negative emotions. The evidence that alexithymia predicts the reproduction variability of sub-second durations of negative affective stimuli corroborates previous neuroimaging studies documenting cerebellar deficits in these individuals.

## Introduction

Research exploring the cognitive and neural correlates of time processing offers a wide amount of evidence supporting the influence of emotions and affective states to the experience of time in both healthy adults and children [1–3]. example, Droit-Volet et al. [4] found that angry faces were judged to last longer than neutral faces. Moreover, watching a scary movie that increases the emotion of fear leads to an overestimation of the duration of neutral stimuli, compared to the exposure to a control condition [5].

A link between emotion processing and time perception is also provided by results from clinical populations affected by affective disorders such as depression, anxiety, and other related clinical conditions. For instance, a meta-analysis documented that depressed patients perceive time to pass less quickly than healthy controls [6]. Mioni et al. [7] found an under-reproduction of supra-second durations in anxiety via time reproduction task, which could indicate an overestimation in duration. Interestingly, this overestimation timing pattern has been reported also in patients affected by post-traumatic stress disorder–PTSD [8, 9].

In summary, this literature suggests that both the incidental modulation of affective states, as well as affective deficits can modulate the way in which the duration of visual stimuli is perceived. A missing knowledge, which would be useful for providing a complete picture of the interaction between time and affective processing, could refers to the role of the emotion processing style as personality trait on the perception of time.

The study of the perception of time in Alexithymia can serve this purpose as it refers to a stable personality feature or trait [10, 11] characterized by an affective-emotional processing disorder that causes difficulties in identifying, describing feelings and emotions [e.g., 12–17]. The study of time estimation in alexithymia would allow to clarify whether a possible influence of emotion on time processing can take place even when we refer to a stable trait of the personality relevant for the processing of emotions, as well as in response to incidental modulation of affective states [4].

A recent study of our group [18] has explored the temporal perspective in Alexithymia using the Zimbardo Time Perspective Inventory [19]. This scale is composed of 56 items, to detect individual differences in time-orientation regarding different aspects of the past, present, and future. The results documented greater attention to past negative aspects in participants with borderline or manifest alexithymia scores, as compared to non-alexithymic individuals. Moreover, the higher the Toronto Alexithymia Scale-20 (TAS-20) score, the greater the tendency to focus on negative aspects of the past and interpret the present in a fatalistic way.

In the current study, we explored the perception of time in alexithymia using a computer-based time reproduction task that involved the temporal estimation of affective stimuli (i.e., facial expressions). We explored the ability to reproduce temporal intervals in the sub-second and supra-second ranges, as any difference between these two scales can be also informative for the neurocognitive correlate associated with the respective timing pattern of this trait. In line with the recent evidence of a rightward bias in alexithymia [20], we predict an overall time overestimation in alexithymia, according with the evidence that a rightward spatial attention bias causes and/or predicts a temporal overestimation [21, 22].

We also expect to detect any influence of negative facial expressions on temporal performance of these individuals according to previous works documenting a perception deficit for negative facial expressions in alexithymia [12, 23]. In this regard, we have chosen the expression of anger for the specific relevance of this emotion in alexithymia. For example, Rosenberg et al. [24] found that alexithymic features are related, in particular, to a lower sensitivity to covert facial expressions of anger.

Finally, we predict greater variability in temporal reproduction in line with the evidence of functional and structural cerebellar abnormalities in alexithymia [24–26], as this neural region is considered important in time processing variability [27].

## Materials and methods

### Participants

31 healthy participants took part in this study (16 females and 15 males, age mean 27.28, Standard Deviation 5.42 age range between 20 and 40 years). Participants were recruited at the department of Cognitive, Psychological, Educational and Cultural Studies, which includes students from different degree courses in social and humanities fields. 16 participants were non-alexithymic; 6 participants were classified as borderline; The remaining 10 participants were classified as alexithymic. Details on how participants were classified in these three groups are provided in the next paragraph. A one-way ANOVA showed no significant difference in gender [*F*(2, 29) = 0, *p* = 0.881, *ηp* 2 = 0.008] and age [*F*(2, 29) = 0,195, *p* = 0.823, *ηp* 2 = 0.013] across the three groups. Most participants were students recruited from the Department of Cognitive, Psychological, Pedagogical, and Cultural Studies (COSPECS) at the University of Messina. Participants were recruited via social network (e.g., Facebook) or email. Exclusion criteria were age over 40 years. All participants gave their written informed consent prior to inclusion in the study and were naïve to its purpose. The data were anonymously collected. The study was approved by the local ethics board (COSPECS Department, University of Messina, Italy) and was conducted in agreement with the principles of the Declaration of Helsinki.

### General procedure

First, the participants were asked to sit down and complete the consent form and the TAS-20. Subsequently, they were asked to perform a trial session of the time reproduction task, to familiarize themselves with the procedure and to have the opportunity to ask the researcher for explanations. After completing the task, participants were allowed to ask questions (if any) about the study goal.

### TAS-20 (20 Item-Toronto Alexithymia Scale)

The TAS-20 is a self-reported scale used to measure alexithymia. It is composed of three subscales: (a) Difficulty in identifying feelings (DIF); (b) Difficulty in describing feelings (DDF); and (c) External oriented thought (EOT). Bagby et al. [28] have proposed three cut-off scores for the classification of individuals: alexithymic subjects (≥61), borderline (score range between 51 to 60), and non-alexithymic subjects (≤50). In this study, the Italian version of the Toronto Alexithymia Scale (20 items, TAS-20) was used, which was validated by Bressi et al. [29] (Cronbach’s alpha 0.75).

### Time reproduction task

We used a version of the time reproduction task previously used in previous studies of our group [29–32]. The test was programmed by using E-prime 2 software. Subjects sat at a distance of 60 cm opposite to the monitor of a pc and were asked to fixate a black cross of 0.2° in diameter, centrally located on the screen. After 500 ms, the stimulus (a photo of a facial expression, size: height, 12 cm, length, 10 cm) appeared in the same location of the fixating cross; after a specified period, it disappeared. Immediately after the stimulus disappeared, subjects had to start reproducing the interval they had just perceived by pressing the space bar on the computer keyboard using the index finger of their dominant hand. When they judged that the same amount of time had elapsed, they had to release the space bar. Facial stimuli included three conditions: i) A neutral expression; ii) A happy expression; iii) A angry expression. These stimuli were selected from the FACES database [33].

Each session consisted in three (one for each expression) separate and consecutive blocks, which were administered in a counterbalanced order. One block consisted of 80 trials in which subjects estimated five sub-second intervals (500, 600, 700, 800, 900 ms) and five supra-second (1500, 1600, 1700, 1800, 1900 ms) intervals presented in randomised order. In all blocks, the inter-trial interval was 2000 ms. No feedback concerning the results of their performance was given to the subjects during the inter-trial interval.

### Data analysis

A preliminary analysis for normality documented a normal distribution for all data (K-Sd = 0.221, p>0.20).

The reproduction performance (reproduction means) was analysed using two separated ANOVA for repeated measures (i.e., one per temporal scale): with GROUP (non-alexithymic vs. borderline/alexithymic subjects), as the between-subject factor, STIMULUS (neutral expression, happy expression, anger expression), and INTERVAL (500–900 ms or 1500–1900 ms) as within-subject factors. The same analysis was applied to reproduction performance variability. As a measure of variability in estimating the presented stimuli, we calculated the coefficient of variation with scores expressed as percentages [CV (Standard Deviation/mean reproduction performance) * 100]. *Post hoc* comparisons were performed using Scheffe test. We also performed a Pearson correlation analysis to explore any relationship between timing performance (i.e., mean and CV) and respective TAS-20 scores. For all statistical analyses, a *p* value of < 0.05 was considered to be significant.

Finally, as further measure of accuracy, we investigated the time reproduction performance of our participants via one sample t-test for single means for both groups and respective stimuli and intervals. Therefore, the reproduction mean provided for each interval was compared with the respective duration to be reproduced. p-level was corrected for corrected for multiple comparisons (i.e., t-test Bonferroni corrected). In this case it was considered significant if **≤** 0.016 (i.e., 0.05/3 types of expressions).

Borderline (score range between 51 to 60) and alexithymic (score > 60) individuals were grouped in a single category, to be compared with no alexithymic individuals, in keeping with previous evidence documenting a similar performance of these two groups in several cognitive and affective tasks [e.g., 12, 18, 20]. Data analysis was performed using Statistica software, version 8.0, StatSoft, Inc., Tulsa, USA.

## Results

### Reproduction means

#### Sub-second

We detected a significant main effect of INTERVAL [*F*_4,112_  =  100.4 *p* < 0.001, *ηp^2^*  =  0.782], showing that the reproduction mean increases with the size of the duration of respective stimuli. No further significant results were found (See supplemental results, S1 Table in S1 File).

#### Supra-second

We detected a significant main effect of INTERVAL [*F*_4,112_  =  64.89 *p* < 0.001, *ηp^2^*  =  0.698], showing that the reproduction mean increases with the size of the duration of respective stimuli. We also found a main effect of the STIMULUS [*F*_2,56_  =  3.516, *p*  =  0.036, *ηp^2^*  =  0.111]. Post-hoc comparison documents temporal overestimation for anger facial expression (M = 1342.6 ± 64.05) compared to neutral facial expression (*M* = 1259.9 ± 59.29, *p* = 0.008), and happy facial expression (M = 1254.2 ± 57.87, p = 0.031). No difference in time reproduction between neutral and happiness expressions was found (p = 0.885). No further significant results were found (See supplemental materials, S2 Table in S1 File).

### Reproduction variability

#### Sub-second

We detected a significant main effect of INTERVAL [*F*_4,112_  =  2.968 *p*  =  0.022, *ηp^2^*  =  0.095], showing that the coefficient of variation decreases with the size of the duration of respective stimuli. However, post hoc comparisons did not show significant difference (p>0.117). No further significant results were found (See supplemental results, S3 Table in S1 File).

#### Supra-second

We detected a significant main effect of INTERVAL [*F*_4,112_  =  3.031 *p*  =  0.020, *ηp^2^*  =  0.097]. post hoc comparisons show significant difference only when comparing CV score in the reproduction of 1600 msec (M = 23.64 ± 1.681) compared with the reproduction of 1800 msec (M = 20.27 ± 1.665, p = 0.033). No further significant results were found (See supplemental results, S4 Table in S1 File).

### Correlation analyses

**Fig 1:**
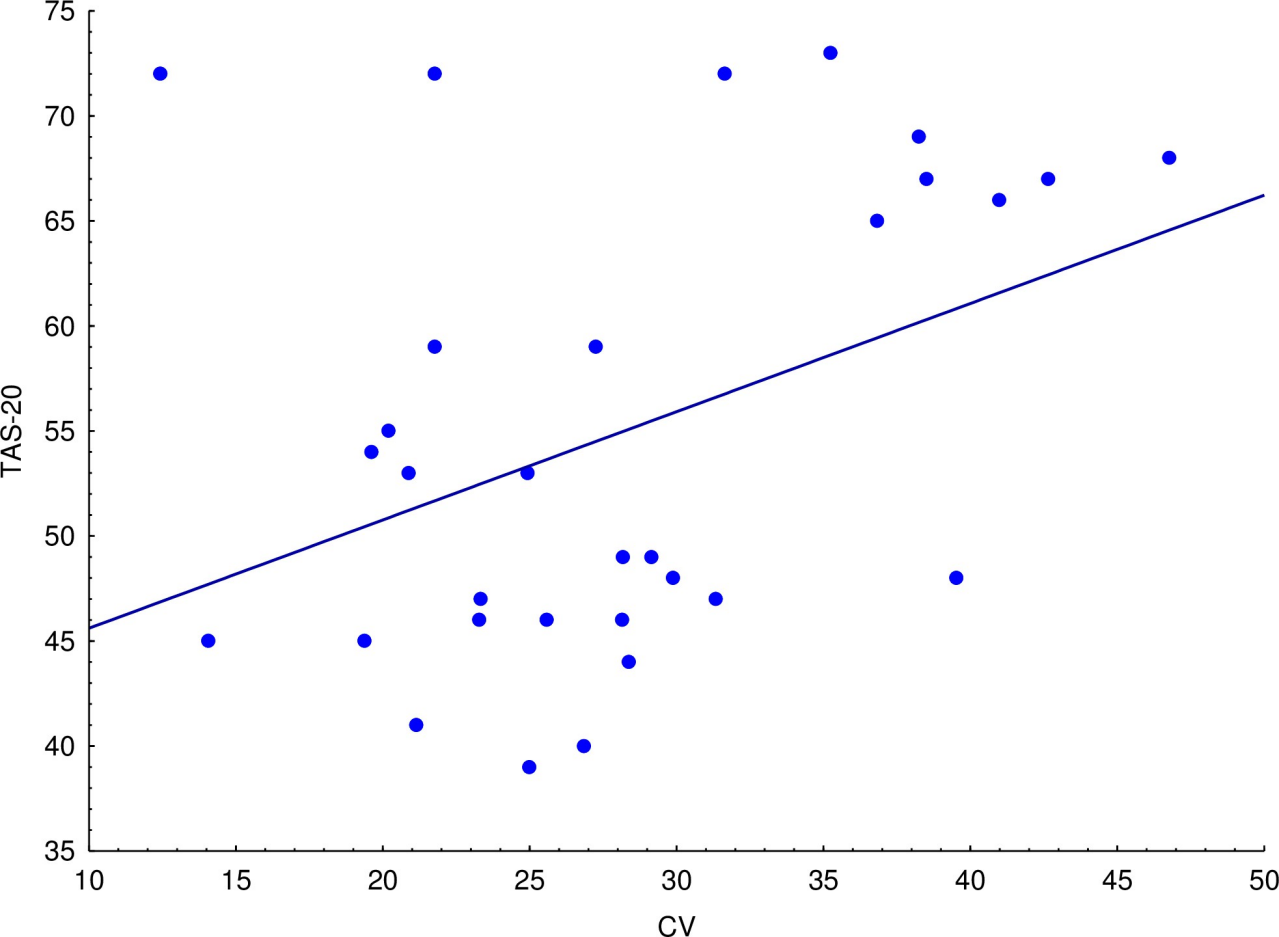
The figure shows a positive correlation between TAS-20 scores and time reproduction variability (coefficient of variation (CV) scores) for anger expression stimuli.

Correlation analyses between reproduction mean and TAS-20 scores did not detect significant results (see Table 1, “mean scores” section for details). On the other hand, we found a positive correlation between TAS-20 and CV scores associated with anger expression stimuli (see Table 1, “CV scores” section and Fig 1 for details).

**Table 1:** Pearson correlation analysis between TAS-20 scores and respective mean reproduction and coefficient of variation (CV) scores.

	*Neutral*	*Happiness*	*Anger*
Mean score			
**Sub-second**	r = 0.019; p = 0.916	r = -0.097; p = 0.601	r = -0.146; p = 0.432
**Supra-second**	r = 0.085; p = 0.646	r = 0.034; p = 0.855	r = -0.088; p = 0.635
	*Neutral*	*Happiness*	*Anger*
CV score			
**Sub-second**	r = 0.170; p = 0.358	r = 0.076; p = 0.682	r = 0.394; p = 0.028*
**Supra-second**	r = 0.248; p = 0.177	r = 0.219; p = 0.235	r = 0.185; p = 0.318

* Indicates significant result.

### One sample t-test for single means

#### Sub-second intervals

Non alexithymic individuals under-reproduced the duration of the neutral expression for all five (500–900 ms) intervals. Moreover, a significant under-reproduction was found for the expression of happiness for the 800 ms interval (Table 2 for details and respective t and p values for all stimuli and intervals).

**Table 2:** One sample t-test analysis for single means (500–900 ms) of the time reproduction of non-alexithymic and borderline/alexithymic individuals for the three facial expressions.

*Non-alexithymic participants*	*Mean*	*Std*.*Dv*.	*N*	*t-value*	*df*	*p-level*	*Cohen’s d*
500 (Neutral)	404.9	162.1	15	-2.271	14	0.039	0.218
500 (Happiness)	512.1	212.2	15	0.221	14	0.828	0.057
500 (Anger)	466.7	130.3	15	-0.987	14	0.340	0.255
600 (Neutral)	471.9	205.1	15	-2.417	14	0.029	0.624
600 (Happiness)	564.1	204.0	15	-0.680	14	0.507	0.175
600 (Anger)	526.5	149.4	15	-1.903	14	0.077	0.491
*700 (Neutral)	532.6	202.2	15	-3.205	14	0.006	0.827
700 (Happiness)	596.5	206.7	15	-1.937	14	0.073	0.500
700 (Anger)	677.4	211.3	15	-0.413	14	0.685	0.106
*800 (Neutral)	591.3	231.0	15	-3.497	14	0.003	0.903
800 (Happiness)	683.6	197.1	15	-2.284	14	0.038	0.589
800 (Anger)	707.5	218.9	15	-1.634	14	0.124	0.422
*900 (Neutral)	704.8	228.8	15	-3.301	14	0.005	0.852
900 (Happiness)	774.1	273.5	15	-1.781	14	0.096	0.460
900 (Anger)	793.9	244.4	15	-1.680	14	0.115	0.433
***Borderline/alexithymic participants***	*Mean*	*Std*.*Dv*.	*N*	*t-value*	*df*	*p-level*	*Cohen’s d*
500 (Neutral)	498.7	216.4	16	-0.023	15	0.981	0.005
500 (Happiness)	461.0	175.3	16	-0.887	15	0.388	0.221
500 (Anger)	509.6	212.0	16	0.182	15	0.857	0.045
600 (Neutral)	551.5	216.1	16	-0.896	15	0.384	0.224
600 (Happiness)	547.8	214.6	16	-0.972	15	0.346	0.243
600 (Anger)	556.3	186.0	16	-0.939	15	0.362	0.234
700 (Neutral)	652.0	202.5	16	-0.947	15	0.358	0.236
700 (Happiness)	594.5	206.2	16	-2.045	15	0.058	0.511
700 (Anger)	666.6	242.3	16	-0.551	15	0.589	0.137
800 (Neutral)	692.0	218.3	16	-1.978	15	0.066	0.004
*800 (Happiness)	639.5	211.2	16	-3.037	15	0.008	0.759
800 (Anger)	722.6	241.5	16	-1.280	15	0.219	0.320
900 (Neutral)	782.7	284.2	16	-1.650	15	0.119	0.412
900 (Happiness)	772.8	264.6	16	-1.921	15	0.073	0.480
900 (Anger)	820.9	296.0	16	-1.067	15	0.302	0.266

* Indicates significant result.

**Fig 2:**
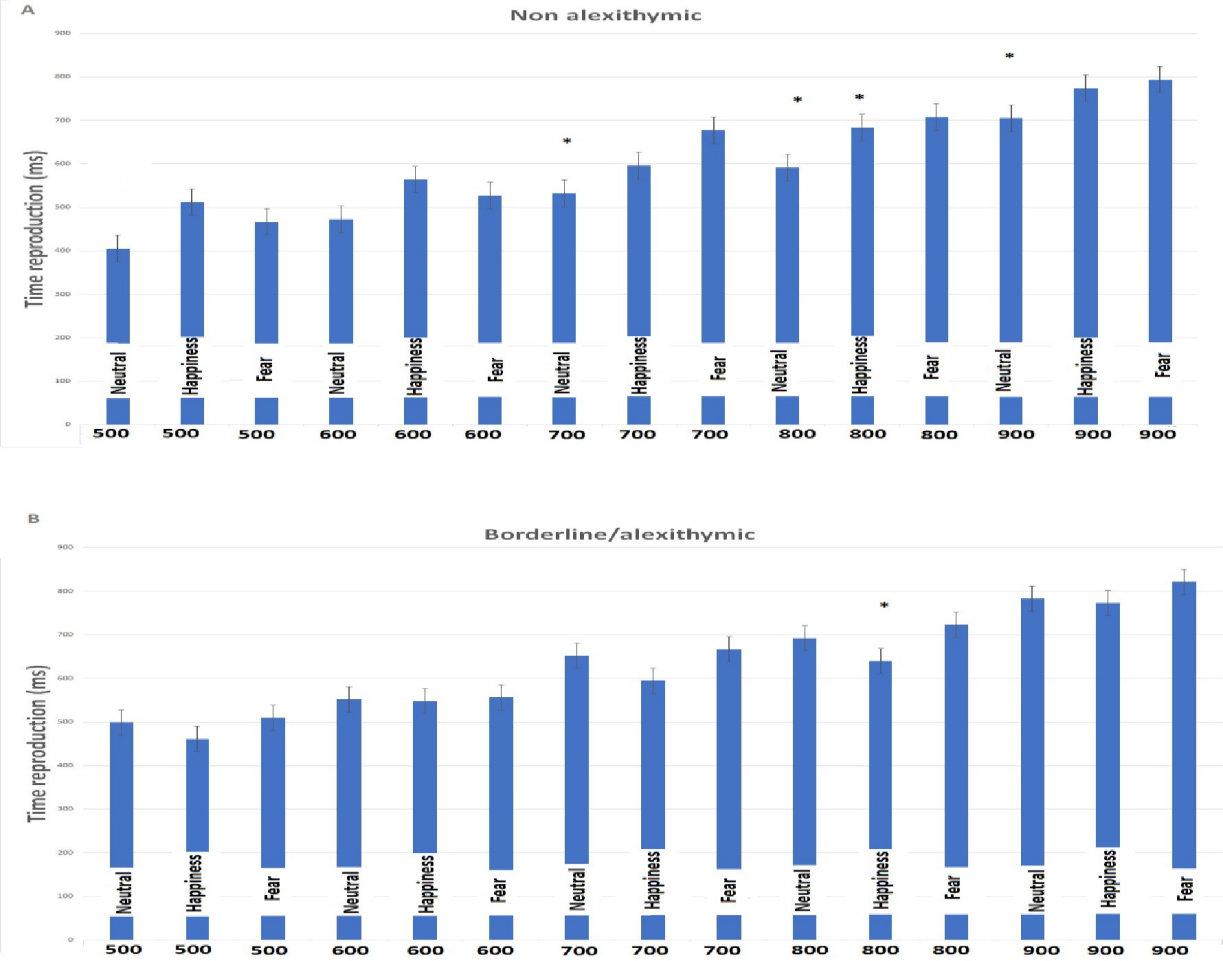
The figure provides time reproduction means for each facial expression and the respective temporal interval. * Indicates significant difference for one sample t-test for single means. Vertical bars denote ± standard error of means.

Borderline/alexithymic individuals under-reproduced the duration of the happiness expression only for the 800 ms interval, as well as the control group. The Table 2 shows mean standard deviation (Std.DV) and respective t and p values for all stimuli and temporal intervals. More details are also provided in the Fig 2.

### Supra-second intervals

Both non alexithymic and borderline/alexithymic individuals under-reproduced the duration of the presented stimuli for all intervals and expressions. See the S5A and S5B Tables in S1 File, supplemental materials, for details.

## Discussion

In the present study we explored time reproduction in healthy individuals with borderline/alexithymic traits and compared the respective performance with non alexithymic individuals. We focused on this personality trait as we aimed to investigate whether (and how) alterations of emotion processing style could affect the subjective ability to keep track of time. Participants were asked to reproduce the durations of emotion related (or control) visual stimuli, such as different types of facial expressions, to investigate any specific interaction between the type (i.e., positive, negative) of emotion and the level of difficulty in processing/recognizing affective stimuli as indicated by the respective TAS-20 score [12].

Overall, the results document a positive correlation between TAS-20 scores and the variability in time reproduction of anger facial expressions. This result, which reflects how repeated responses are scattered from their target [34–36] suggests an altered scalar property of time in borderline/alexithymic individuals for negative emotional stimuli. The concept of scalar property refers to the way in which the standard deviation and mean of the participants’ response distribution covary [36]. In this regard, CV is considered the golden measure to explore this property of the timing ability [37]. Our results suggest that variability in temporal reproduction of sub-second durations increases with the severity of the alexithymia personality trait. The evidence of a selective bias for anger expression is in line with previous works suggesting a specific relevance of anger expression in Alexithymia [24, 38–40]. For example, results from a visual ERP study [39] suggest that the perception of anger facial expression is disrupted in alexithymia. Given the relevance of decision-making processes for the execution of the proposed timing task, our results provide further evidence to the hypothesis of impaired integration of social anger signals into judgemental processes in alexithymia [24].

The evidence that the variability in the reproduction sub-second durations of anger facial expressions increases with the severity of alexithymia suggests a possible involvement of the cerebellum. CV is a measure particularly sensitive to alteration in cerebellar activity [41–44]. For example, Gibbon et al. [36] reported increased scalar variability of time in cerebellar patients. Neuroimaging evidence [25–27] have also reported a direct relationship between alexithymia and cerebellar volume. The evidence of a selective involvement of the sub-second range of durations further supports the cerebellar hypothesis, in line with the suggestion that the cerebellum is specialized in the representation of sub-second durations [43, 44], contrary to the timing of supra-second durations, which mainly relates with the cortical activity [45–47] Finally, the potential role of the cerebellum in explaining the current result is corroborated by the emerging literature documenting its involvement in social cognition and emotion processing [57]. Importantly, the study by Ferrucci et al. [48] showed a selective involvement of the cerebellum in the emotional recognition of anger, which is the facial expression for which we found the correlation pattern between TAS-20 and time reproduction.

Another noteworthy finding is the tendency of borderline/alexithymic participants to overestimate sub-second durations, compared to non-alexithymic participants. Non-alexithymic individuals underestimate sub-second durations (i.e., the 700–900 ms range) of neutral stimuli, and this difference is statistically significant, as shown via one sample t-test analysis. On the other hand, the difference between the reproduced means and respective durations was found to be not statistically significant in borderline/alexithymic participants. This latter result can be interpreted in terms of temporal overestimation, in line with our initial prediction, linking this timing pattern with the rightward bias in alexithymia [18]. An alternative interpretation for this result is that borderline/alexithymic participants can be more accurate in reproducing sub-second durations, as their temporal performance resulted closer to the respective temporal landmark. However, we think this interpretation is unlikely, in keeping with the evidence of impaired cognitive control in alexithymia [49] which seems to be necessary for accurate timing performance [50].

Finally, it should be mentioned the overall overestimation of durations related to the anger expression, as suggested by the main effect of the stimulus. The block per stimulus interaction term shows that this effect is specific for sub-second intervals. This result confirms previous works [e.g., 4] documenting that the duration of angry faces is judged to last longer than neutral faces.

In conclusion, our study shows that alexithymia, an emotion processing style characterized by an affective-emotional processing disorder that causes difficulties in identifying, describing feelings and emotions, affects time perception. This expands the literature on emotion-time processing interplay by showing that even a stable trait of the personality relevant for the processing of emotions affects the experience of time as well as the incidental modulation of affective states [4]. Moreover, it adds supports to the suggestion on relevance of this cognitive function as a transdiagnostic index for mental disorders [47].

Some limitations of the present study should be mentioned. First, the overall number of participants was relatively low, and no sample size calculation was applied. This might explain why the ANOVA on means and CV scores did not document a significant between groups difference regarding the main research hypotheses. Moreover, we have not collected data on anxiety and interoceptive awareness, which are known to be relevant to the experience of time processing [47, 51].

Future studies involving a larger sample, other type of timing tasks, other negative emotional expressions such as disgust, as there is evidence of altered time perception in clinical disorders such as Anorexia Nervosa [52] and Hyperphagia [53] which are known to be affected by abnormal disgust sensitivity as trait of personality [54–56]. For a review on clinical models see also [57]; and other emotion processing style as personality traits [e.g., see 58–60], are needed to explore in more detail the interplay between affective processing emotional regulation, and the experience of time.

## Supporting information

S1 File Supplementary tables. (DOCX)
