# Supplementary material for: Evidence of abnormal scalar timing property in alexithymia
Source: PLoS One. 2023 Jan 23;18(1):e0278881. doi: 10.1371/journal.pone.0278881 (PMC9870170; doi:10.1371/journal.pone.0278881)

**Supplemental materials**

Table 1. **ANOVA on reproduction mean of sub-second durations** of non-alexithymic and borderline/alexithymic individuals for the three facial expressions.

| Repeated Measures Analysis of Variance with Effect Sizes and Powers (Spreadsheet1) Sigma-restricted parameterization Effective hypothesis decomposition | | | | | | | | |
| --- | --- | --- | --- | --- | --- | --- | --- | --- |
|  | **SS** | **Degr. of - Freedom** | **MS** | **F** | **p** | **Partial eta-squared** | **Non-centrality** | **Observed power (alpha=0,05)** |
| **Intercept** | 161943868 | 1 | 161943868 | 340,0085 | 0,000000 | 0,923915 | 340,0085 | 1,000000 |
| **"500"** | 1144191 | 2 | 572096 | 1,2011 | 0,315883 | 0,079016 | 2,4023 | 0,240804 |
| **Error** | 13336219 | 28 | 476294 |  |  |  |  |  |
| **EMO** | 188822 | 2 | 94411 | 1,3969 | 0,255848 | 0,047519 | 2,7938 | 0,287786 |
| **EMO*"500"** | 486455 | 4 | 121614 | 1,7994 | 0,141827 | 0,113890 | 7,1975 | 0,513971 |
| **Error** | 3784833 | 56 | 67586 |  |  |  |  |  |
| **INTERV** | 4535134 | 4 | 1133783 | 100,4688 | 0,000000 | 0,782048 | 401,8752 | 1,000000 |
| **INTERV*"500"** | 35984 | 8 | 4498 | 0,3986 | 0,919277 | 0,027682 | 3,1887 | 0,180904 |
| **Error** | 1263912 | 112 | 11285 |  |  |  |  |  |
| **EMO*INTERV** | 65685 | 8 | 8211 | 1,6525 | 0,111322 | 0,055730 | 13,2202 | 0,717900 |
| **EMO*INTERV*"500"** | 75726 | 16 | 4733 | 0,9526 | 0,510033 | 0,063706 | 15,2410 | 0,638931 |
| **Error** | 1112956 | 224 | 4969 |  |  |  |  |  |

Table 2. **ANOVA on reproduction mean of supra-second durations** of non-alexithymic and borderline/alexithymic individuals for the three facial expressions.

| Repeated Measures Analysis of Variance with Effect Sizes and Powers (Spreadsheet1) Sigma-restricted parameterization Effective hypothesis decomposition | | | | | | | | |
| --- | --- | --- | --- | --- | --- | --- | --- | --- |
|  | **SS** | **Degr. of - Freedom** | **MS** | **F** | **p** | **Partial eta-squared** | **Non-centrality** | **Observed power (alpha=0,05)** |
| **Intercept** | 669398357 | 1 | 669398357 | 517,8609 | 0,000000 | 0,948705 | 517,8609 | 1,000000 |
| **"500"** | 2492760 | 2 | 1246380 | 0,9642 | 0,393580 | 0,064435 | 1,9285 | 0,200286 |
| **Error** | 36193414 | 28 | 1292622 |  |  |  |  |  |
| **EMO** | 661096 | 2 | 330548 | 3,5161 | 0,036434 | 0,111566 | 7,0323 | 0,632478 |
| **EMO*"500"** | 630266 | 4 | 157567 | 1,6761 | 0,168391 | 0,106919 | 6,7043 | 0,482038 |
| **Error** | 5264507 | 56 | 94009 |  |  |  |  |  |
| **INTERV** | 3082008 | 4 | 770502 | 64,8984 | 0,000000 | 0,698595 | 259,5937 | 1,000000 |
| **INTERV*"500"** | 158651 | 8 | 19831 | 1,6704 | 0,113354 | 0,106594 | 13,3630 | 0,705789 |
| **Error** | 1329713 | 112 | 11872 |  |  |  |  |  |
| **EMO*INTERV** | 40675 | 8 | 5084 | 0,4038 | 0,917686 | 0,014217 | 3,2306 | 0,188496 |
| **EMO*INTERV*"500"** | 168133 | 16 | 10508 | 0,8346 | 0,645408 | 0,056261 | 13,3539 | 0,564983 |
| **Error** | 2820283 | 224 | 12591 |  |  |  |  |  |

**Table 3. ANOVA on Coefficient of variation sub-second durations** of non-alexithymic and borderline/alexithymic individuals for the three facial expressions.

| Repeated Measures Analysis of Variance with Effect Sizes and Powers (Spreadsheet1) Sigma-restricted parameterization Effective hypothesis decomposition | | | | | | | | |
| --- | --- | --- | --- | --- | --- | --- | --- | --- |
|  | **SS** | **Degr. of - Freedom** | **MS** | **F** | **p** | **Partial eta-squared** | **Non-centrality** | **Observed power (alpha=0,05)** |
| **Intercept** | 321067,2 | 1 | 321067,2 | 452,4813 | 0,000000 | 0,941725 | 452,4813 | 1,000000 |
| **"500"** | 2765,7 | 2 | 1382,9 | 1,9489 | 0,161277 | 0,122195 | 3,8977 | 0,369166 |
| **Error** | 19868,0 | 28 | 709,6 |  |  |  |  |  |
| **EMO** | 284,2 | 2 | 142,1 | 1,0200 | 0,367211 | 0,035147 | 2,0399 | 0,219248 |
| **EMO*"500"** | 1339,8 | 4 | 334,9 | 2,4045 | 0,060333 | 0,146574 | 9,6179 | 0,654659 |
| **Error** | 7800,7 | 56 | 139,3 |  |  |  |  |  |
| **INTERV** | 1309,8 | 4 | 327,5 | 2,9686 | 0,022557 | 0,095857 | 11,8742 | 0,778312 |
| **INTERV*"500"** | 685,0 | 8 | 85,6 | 0,7763 | 0,624361 | 0,052536 | 6,2104 | 0,344975 |
| **Error** | 12354,4 | 112 | 110,3 |  |  |  |  |  |
| **EMO*INTERV** | 649,5 | 8 | 81,2 | 1,0833 | 0,375683 | 0,037250 | 8,6667 | 0,498302 |
| **EMO*INTERV*"500"** | 968,6 | 16 | 60,5 | 0,8078 | 0,676124 | 0,054552 | 12,9248 | 0,547234 |
| **Error** | 16786,6 | 224 | 74,9 |  |  |  |  |  |

Table 4. **ANOVA on Coefficient of variation sub-second durations** of non-alexithymic and borderline/alexithymic individuals for the three facial expressions.

| Repeated Measures Analysis of Variance with Effect Sizes and Powers (Spreadsheet1) Sigma-restricted parameterization Effective hypothesis decomposition | | | | | | | | |
| --- | --- | --- | --- | --- | --- | --- | --- | --- |
|  | **SS** | **Degr. of - Freedom** | **MS** | **F** | **p** | **Partial eta-squared** | **Non-centrality** | **Observed power (alpha=0,05)** |
| **Intercept** | 195796,6 | 1 | 195796,6 | 259,3517 | 0,000000 | 0,902558 | 259,3517 | 1,000000 |
| **"500"** | 1389,1 | 2 | 694,6 | 0,9200 | 0,410232 | 0,061662 | 1,8400 | 0,192800 |
| **Error** | 21138,5 | 28 | 754,9 |  |  |  |  |  |
| **EMO** | 45,2 | 2 | 22,6 | 0,2928 | 0,747338 | 0,010347 | 0,5855 | 0,094193 |
| **EMO*"500"** | 68,7 | 4 | 17,2 | 0,2225 | 0,924737 | 0,015647 | 0,8902 | 0,094586 |
| **Error** | 4324,2 | 56 | 77,2 |  |  |  |  |  |
| **INTERVS** | 544,1 | 4 | 136,0 | 3,0318 | 0,020447 | 0,097700 | 12,1273 | 0,788053 |
| **INTERVS*"500"** | 112,1 | 8 | 14,0 | 0,3123 | 0,959991 | 0,021823 | 2,4987 | 0,147510 |
| **Error** | 5025,3 | 112 | 44,9 |  |  |  |  |  |
| **EMO*INTERVS** | 410,8 | 8 | 51,4 | 1,1526 | 0,329258 | 0,039538 | 9,2212 | 0,528686 |
| **EMO*INTERVS*"500"** | 700,8 | 16 | 43,8 | 0,9831 | 0,476167 | 0,065612 | 15,7292 | 0,656849 |
| **Error** | 9980,0 | 224 | 44,6 |  |  |  |  |  |

**Table 5A.** One sample t-test analysis for single means of the time reproduction of non-alexithymic individuals for the three facial expressions.


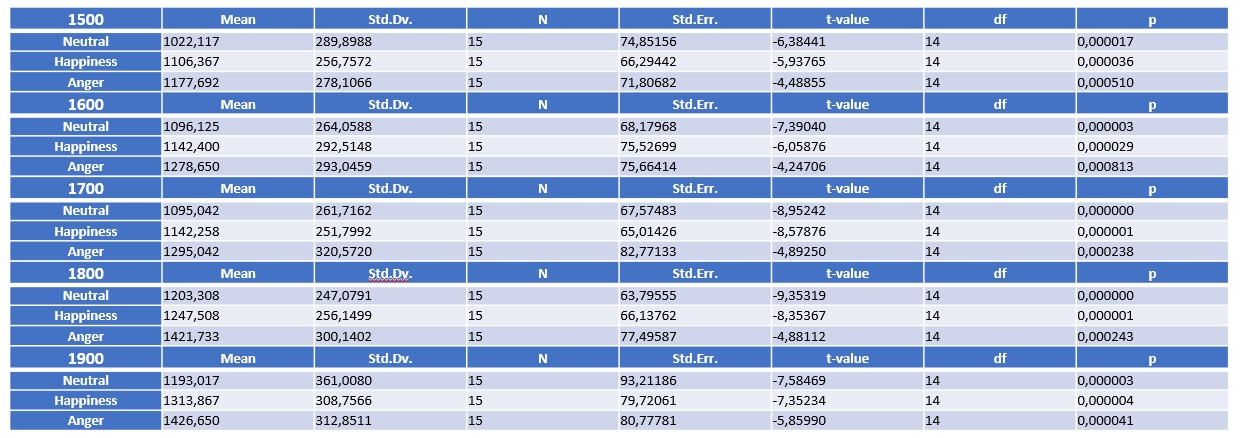


**Table 5B.** One sample t-test analysis for single means of the time reproduction of borderline/alexithymic individuals for the three facial expressions.


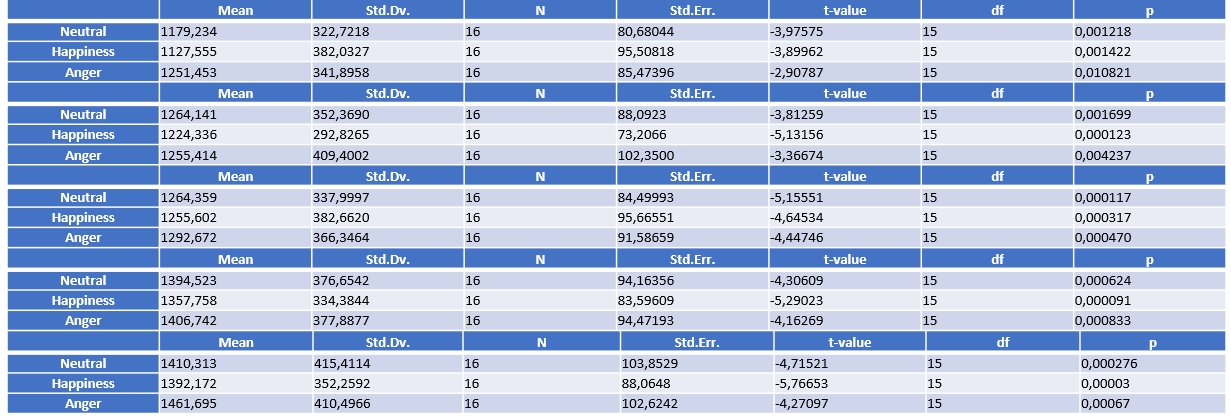

Supplement: S1 File — (DOCX) [file pone.0278881.s001.docx]
